# Effect of sexual maturation on muscle gene expression of rainbow trout: RNA-Seq approach

**DOI:** 10.1002/phy2.120

**Published:** 2013-10-23

**Authors:** Mohamed Salem, Meghan L Manor, Aunchalee Aussanasuwannakul, Patrick Brett Kenney, Gregory M Weber, Jianbo Yao

**Affiliations:** 1 Department of Biology, Middle Tennessee State UniversityMurfreesboro, Tennessee, 37132; 2 Division of Animal and Nutritional Science, West Virginia UniversityMorgantown, West Virginia, 26506-6108; 3 Institute of Food Research and Product Development, Kasetsart UniversityBangkok, 10900, Thailand; 4 The National Center for Cool and Cold Water Aquaculture, USDA Agricultural Research ServiceLeetown, West Virginia, 25430

**Keywords:** muscle, rainbow trout, reproduction, RNA-Seq

## Abstract

Muscle degradation occurs as a response to various physiological states that are regulated by specific molecular mechanisms. Previously, we characterized the metabolic changes of muscle deterioration of the female rainbow trout at full sexual maturity and spawning (Salem et al., *Physiol. Genomics* 2006;28:33–45; *J. Proteomics* 2010;73:778–789). Muscle deterioration in this model represents nutrient mobilization as a response to the energetic overdemands of the egg/ovarian growth phase. Our recent studies showed that most of the changes in muscle growth and quality start 2–3 months before spawning. Gravid fish exhibited reduced intramuscular fat that is lower in saturated and monounsaturated fatty acids and higher in polyunsaturated fatty acids compared to sterile fish. In this study, RNA-Seq was used to explain the mechanisms underlying changes during this phase of sexual maturity. Furthermore, to minimize changes due to nutrient deficits, fish were fed on a high-plane of nutrition. The RNA-Seq technique identified a gene expression signature that is consistent with metabolic changes of gravid fish. Gravid fish exhibited increased abundance of transcripts in metabolic pathways of fatty acid degradation and up-regulated expression of genes involved in biosynthesis of unsaturated fatty acids. In addition, increased expression of genes involved in the citric acid cycle and oxidative phosphorylation was observed for gravid fish. This muscle transcriptomic signature of fish fed on a high nutritional plane is quite distinct from that previously described for fish at terminal stages of maturity and suggest that female rainbow trout approaching spawning, on high nutritional planes, likely mobilize intramuscular fat rather than protein to support gonadal maturation.

## Introduction

Skeletal muscle is the most abundant and edible tissue of fish and typically makes up more than 50% of the fish weight (Salem et al. 2006a; Aussanasuwannakul et al. 2011). Muscle development, growth, and quality are complex and polygenic traits; regulated by synchronized expression of several genes that are organized in transcriptional networks (Gerrard 2003; Salem et al. 2008). In addition, the molecular regulation of lipid accumulation within fish muscle deserves investigation due to the important impact of fish lipids on human health and fillet quality attributes, particularly flavor, texture, and shelf-life (Manor et al. 2012).

Several transcriptome-wide expression studies have dealt with muscle growth and quality and identified candidate genes and physiological pathways to target in hypothesis-driven studies (Reecy et al. 2006). Microarray studies identified genes that control muscle development, growth and quality in model organisms including mice (Raffaello et al. 2006), humans (Timmons and Sundberg 2006) cattle (Byrne et al. 2005) and swine (Cagnazzo et al. 2006). Additionally, gene expression studies have shown that various types of skeletal muscle degeneration follow a common sequence of changes in gene expression (Lecker et al. 2004). In mammals, despite different signals stimulating muscle degradation, the molecular changes of the degenerating muscle share many common cascades. In fish, some studies investigated effects of nutrition (Rescan et al. 2007; Bower and Johnston 2010), growth hormone (Gahr et al. 2008), insulin, and insulin-like growth factor-I (Cleveland and Weber 2010; Seiliez et al. 2011) and spawning migration (Miller et al. 2009) on muscle. However, the molecular networks which control muscle degeneration have not been intensively described. Data previously produced in our laboratory indicate that changes in gene expression of trout muscle atrophy (at terminal stages of sexual maturity) are, in general, similar to mammalian muscle atrophy alterations. These changes cause an arrest of normal cell growth, anaerobic respiration, and protein biosynthesis and an increase in protein degradation (Salem et al. 2006a,b, 2010a). Nevertheless, other changes/mechanisms unique to fish were observed. For example, expression of the proteasome proteolytic pathway, which is largely responsible for mammalian main part of proteolysis was not affected in our study of sexual maturation-induced trout muscle degradation (Salem et al. 2006a,b). In addition, contradicting the general trend of suppressing the mitochondrial energy production reported in mammalian muscle catabolic states of mammalian muscle degradation, our microarray studies of trout demonstrated an enhanced competence for fish aerobic ATP production, buffering and utilization during sexual maturation-induced trout muscle degradation (Salem et al. 2006b).

Previously, we have used transcriptomic, proteomic, and target gene/pathway approaches to examine genetic contributions to muscle growth and fillet quality in rainbow trout (Salem et al. 2006a,b, 2008, 2010a; Wang et al. 2011). Numerous candidate genes that are linked to changes in fish muscle growth and fillet quality have been detected. In addition, muscle deterioration, as a physiological response to the elevated energetic needs of sexual maturation, was described as a distinct model for examining mechanisms of muscle degeneration in fish (Salem et al. 2006b, 2010a). Using microarray-chip and proteomic technologies, we studied the problem of muscle quality deterioration in gravid female rainbow trout compared to sterile (triploid [3N]) female fish (Salem et al. 2006b, 2010a). Most of the muscle deterioration observed in this model was the result of nutrient mobilization in response to homeorhetic, energy demands during later stages of egg/ovarian development and spawning. More recent studies in our laboratory showed that most of the changes in muscle growth and quality start 2–3 months before spawning (Aussanasuwannakul et al. 2011, 2012; Manor et al. 2012).

The objective of this study was to investigate effects of sexual maturation on gene expression in muscle of female rainbow trout during the 2–3 months phase preceding the full sexual maturity and spawning. Furthermore, to minimize changes due to nutrient (at least energy) deficits, fish were fed on a high plane of nutrition. Availability of RNA-Seq technology provides new opportunities for researchers, allowing accurate and affordable, global gene expression studies (Marguerat and Bahler 2010). The field of RNA-Seq gene profiling is still relatively new but rapidly growing in fish (Jeukens et al. 2010; Huang et al. 2011, 2012; Oleksiak et al. 2011; Li et al. 2012; Sun et al. 2012). In this study, we used the high-throughput gene expression analysis, RNA-Seq, to measure gene expression patterns that are associated with changes in percentage of separable muscle, muscle composition and quality. The long-term goal of this work is to decipher the molecular mechanisms that control fish muscle growth and quality.

## Material and Methods

### Fish and sampling

Fish used in this study were previously described (Aussanasuwannakul et al. 2011; Manor et al. 2012). Briefly, two families, each containing diploid (2N) and 3N rainbow trout, were generated and maintained at the National Center for Cool and Cold Water Aquaculture (NCCCWA; U.S. Department of Agriculture, Agricultural Research Service, Leetown, WV). Fish were confirmed as 2N or 3N by flow cytometry (Hershberger and Hostuttler 2007; Allen 1983). Animals were fed a commercial feed, Zeigler GOLD Floating 5.0 mm pelleted feed (42% protein, 16% fat, and 2% fiber; 316520-36-44; Zeigler Brothers, Inc., Gardners, PA), throughout the course of the experiment. Fish were fed on a tank-by-tank basis. Part of the daily ration was delivered by belt feeder (minimum of 0.8% of body weight). At the end of the day, fish were fed by hand to apparent satiation. The amount of feed delivered by the belt feeder was altered depending on appetite. Fish were initially maintained as part of stocks in five, 1.22 m-diameter tanks. In July, each of the five tanks was stocked with 35 fish, totaling 175 fish for this study. The 35 fish assigned to each tank consisted of 2N and 3N fish from each of the two families. In July, September, November, and December 2008; and January and March 2009 fish were shifted to a different tank to reduce biases associated with tank. Most fish were shifted as a group but some fish were moved separately to make tank densities equal. Similar tank densities were maintained during the study. To avoid temperature effects, water temperatures were maintained between 12.0 and 13.5°C. A simulated ambient photoperiod was maintained with artificial lighting. Passive integrated transponders (Avid Identification Systems Inc., Norco, CA) were implanted in the musculature below the dorsal fin as tags for individual fish identification. Fish care and experimentation followed the guidelines outlined by the U.S. Department of Agriculture (USDA) and NCCCWA Animal Care and Use Committee, which are in line with the National Research Council publication Guide for Care and Use of Laboratory Animals.

Five fish from each of two family by two ploidy (2N, fertile; and 3N, sterile) combinations (five fish × four combinations = 20 fish) were sampled at two points, December 2008 and January 2009 (40 fish in total). These sampling points represented fish approaching spawning at age endpoints of 21 and 22 months, respectively. Fish were held off feed 24 h prior to sampling and were anesthetized using an overdose of MS222 (Western Chemicals, Ferndale, CA) at 300 mg/L. Gravimetric measurements are reported in Manor et al. (2012) and Aussanasuwannakul et al. (2011). Gonado-somatic index (GSI) was calculated as GSI = (*W*_G_/*W*_B_) × 100, where *W*_G_ is gonad weight and *W*_B_ is whole body weight in grams. Fertile fish were gravid, with an average GSI of 9.4 ± 0.03 (*n* = 20). The GSI of sterile fish was 0.03 ± 0.45 (*n* = 20).

### Muscle yield and quality analysis

Separable muscle data were collected and fillet yield and quality analyses were conducted as previously described (Manor et al. 2009; Aussanasuwannakul et al. 2011). Separable muscle weight was calculated as a percent of whole body weight. A 40 × 80 mm muscle section was separated, parallel to the long axis of the body, from the dorsal musculature for texture analysis (data reported in Aussanasuwannakul et al. 2011). The remaining muscle from the fillets was pulverized with liquid nitrogen in a Waring Blender (Waring, New Hartford, CT) and kept at −25°C for chemical composition analyses.

Proximate composition of muscle was determined using AOAC (1990)-approved methods. Crude fat was analyzed using the Soxhlet solvent extractor with petroleum ether. Moisture was determined by the oven-drying method (100°C for 18 h). Kjeldahl nitrogen was determined (AOAC 1990) and nitrogen content was multiplied by 6.25 as the conversion factor to calculate percent crude protein.

Texture of fillet sections was determined using a five-blade, Allo-Kramer shear cell attached to a Texture Analyzer (Model TA-HDi®; Texture Technologies Corp., Scarsdale, NY), equipped with a 50-kg load cell and at a crosshead speed of 127 mm/min. Force-deformation graphs were recorded and analyzed using the Texture Expert Exceed software (version 2.60; Stable Micro Systems Ltd., Surrey, U.K.). Peak shear force (g/g sample) was recorded.

### Fatty acid analyses

Total lipids were extracted according to Bligh and Dyer (1959) using a chloroform–methanol mixture (2:1 v/v). Due to variation in tissue crude fat content, as determined from proximate analyses, the amount of tissue extracted varied so that a standard amount of fat (35 mg) was extracted for each sample. Fatty acids were methylated using the method described by Fritsche and Johnston (1990). Fatty acid methyl esters (FAME) were quantified using a Varian CP-3800 Gas Chromatograph (Varian Analytical Instruments; Walnut Creek, CA) equipped with a flame ionization detector (FID). A wall-coated, open-tubular (WCOT) fused silica capillary column (100-m length, 0.25-mm inside diameter; Varian Inc.) was used to separate FAME. The stationary phase was CP-Sil 88, and nitrogen was the carrier gas at a flow of 0.3 mL/min. A 10–1 split ratio was applied for all samples. An oven temperature of 140°C for 5 min followed by a temperature ramp of 3°C/min to 235°C was used; 235°C was held for 15 min. The total separating time per sample was 68.5 min. Injector (11–77 injector, Varian Inc.) and detector (FID, Varian Inc.) temperatures were maintained at 270 and 300°C, respectively. FAMEs were identified based on comparison to retention times of standard FAMEs (SupelcoTM quantitative standard FAME 37; Sigma-Aldrich, St. Louis, MO). Peak area and relative amount of each fatty acid were computed by an integrator using the Star GC workstation version 6 software (Varian Inc.). Fatty acids are reported as percent relative peak areas. Complete fatty acid profiles of muscle, liver, visceral adipose tissue, ovaries, and diet are reported in Manor et al. (2012).

### RNA-Seq and qPCR analysis

RNA-Seq analyses were carried out using muscle tissue from 10 gravid and 10 sterile female rainbow trout collected in December 2008 and January 2009 (a total of 40 fish). Muscle tissues, collected at NCCCWA, were flash frozen in liquid nitrogen, shipped on dry ice to West Virginia University, and stored at −80°C until total RNA isolation. Total RNA was isolated from each sample using TRIzol™ (Invitrogen, Carlsbad, CA). Due to cost constraints, equal amounts of total RNA from 10 muscle samples within each time by ploidy group were pooled and used for RNA-Seq sequencing (a total of four RNA-Seq samples, two ploidies × 2 months).

cDNA libraries were prepared and sequenced on an Illumina Genome Analyzer (single-end, 36 bp read length) at the National Center for Genome Resources (Santa Fe, NM) as previously described (Salem et al. 2012). CLC Genomics Workbench (CLC bio, Aarhus, Denmark) was used for RNA-Seq analysis to map/count sequence reads to a previously assembled reference transcriptome (Salem et al. 2010b). RPKM (reads per kilo base per million) values were calculated using CLC Genomics Workbench. Functional annotation of the differentially expressed genes was carried out by BLASTx (Basic Local Alignment Search Tool) search against the NCBI nonredundant protein database using the Blast2GO suite (Götz et al. 2008). BLAST result accessions and GO terms were used to retrieve KEGG (Kyoto Encyclopedia of Genes and Genomes) pathway maps. Quantitative real-time polymerase chain reaction (RT-PCR) was used to confirm expression of a subset of five differentially expressed genes identified by RNA-Seq using the individual samples used for the RNA-Seq experiment as we previously described (Salem et al. 2006b, 2007). Real-time PCR primers were designed based on each gene sequence (Table 1).

**Table 1:** Primers used for real-time RT-PCR analysis.

Gene name	Forward primer	Reverse primer	GenBank acc#
GAPDH	5-TTGTAAAGCCCCTGTTCTGG-3	5-GAAGCAGGTTCAGTGCAACA-3	EZ905446
EL	5-CCAGTACAGCTGGAGGAAGC-3	5-TCGTTGTTCATCAGCGAGTC-3	CU063634
ACAA2	5-CAGACACAGCAGAGGTGGAA-3	5-ATGGGTACCTTGCCCTTCTT-3	EZ791335
HADHB	5-CCTATAAGGCTGAGGCGTTG-3	5-TTCGTCACAGCAGGAAGATG-3	EZ906059
EHHADH	5-GAAGCTGCAGGTGTTTAGGC-3	5-GATGTCCAAGCCAGAGGTGT-3	EZ838632
β-Actin	5-GCCGGCCGCGACCTCACAGACTAC-3	5-CGGCCGTGGTGGTGAAGCTGTAAC-3	AJ438158

### Statistical analyses

For the phenotypic data analyses, the experiment was conducted in the context of a 2 × 2 × 2 randomized complete block design with a fixed block effect (family) at two endpoints (December 2008; and January 2009) and for two sex conditions (2N and 3N). Five fish were randomly assigned to each of four treatment combinations (two ploidies × two families) at each endpoint.

Muscle yield and fillet composition data were analyzed using the PROC MIXED procedure of SAS® system for Windows, version 9.1(SAS Institute Inc., 2004). Variance components were estimated by restricted maximum likelihood method (Ramon et al. 2006) for testing fixed effects which included ploidy. The DDFM = KR option was used to invoke an adjustment to standard errors and test statistics and the degree of freedom approximation. The DDFM = KR option specifies the Kenward-Roger method for computing the denominator degrees of freedom for the fixed effects. The PDIFF function of LSMeans was used to perform pair-wise comparisons. PDIFF requests that the differences between least square means of the factor levels are tested against 0 and *P*-values be printed. Significant differences were defined at *P* ≤ 0.05.

For the RNA-Seq analysis, RPKM values were analyzed for statistical significance using CLC Genomics Workbench Gaussian-based tests. Differential gene expression between groups “Gravid” and “sterile” (each represented by two samples; December [21 M] and January [22 M]) was determined to be statistically significant by Student's *t* test at fold change ≥±2 and a false discovery rate (FDR) ≤0.01.

## Results and Discussion

### Muscle compositional and physical response to sexual maturation

**Figure 1:**
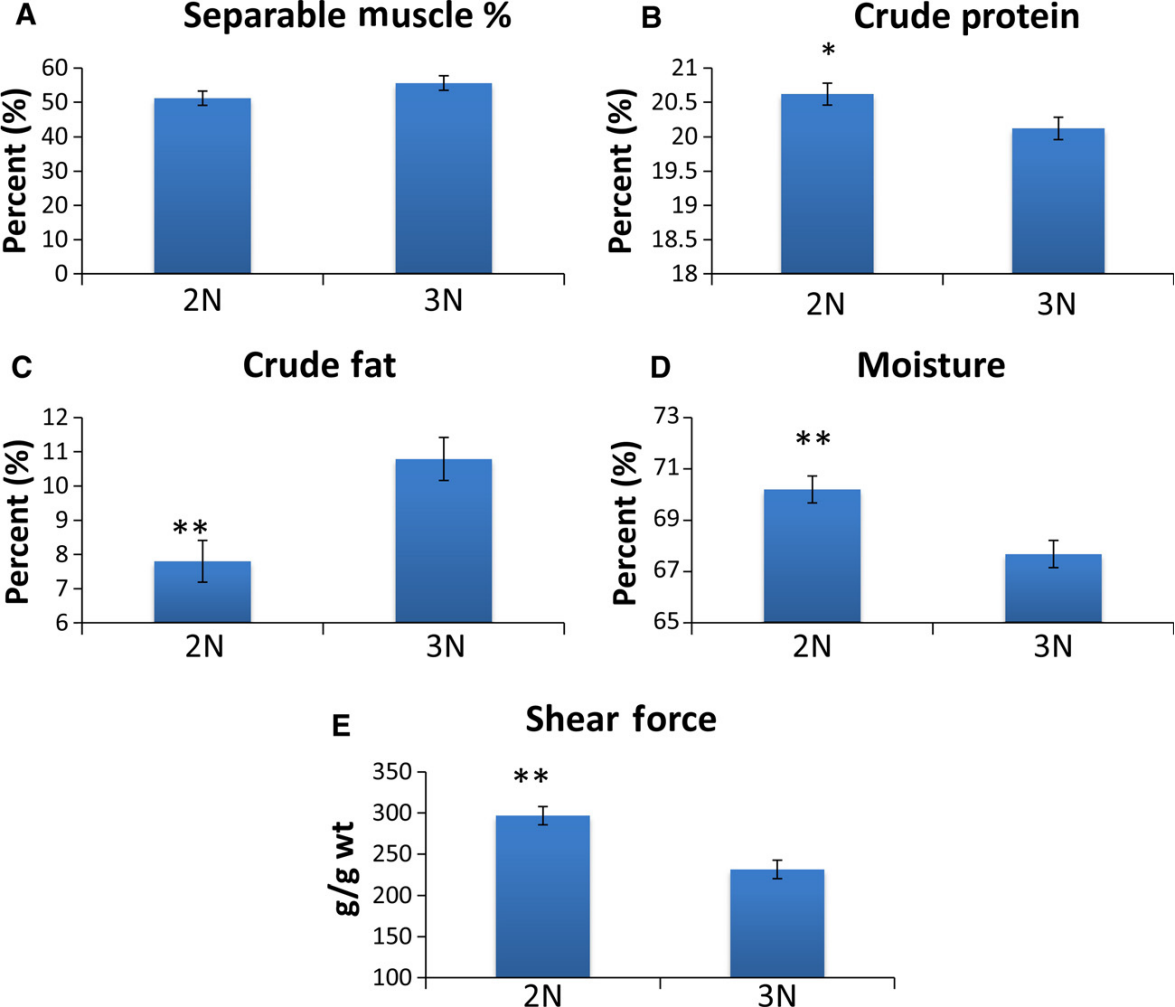
Effects of sexual maturation on female rainbow trout separable muscle percentage (A), total protein (B), fat content (C), moisture (D) and muscle shear force (E). * and ** indicate the *P* values <0.05 and <0.01, respectively, (LSMeans + SEM, *N* = 20). 2N and 3N indicate diploid and triploid fish, respectively.

Gravid fish in December and January yielded a numerically lower percentage of separable muscle compared to sterile 3N fish; 51.2 ± 2.1% and 55.6 ± 2.1%, respectively (Fig. 1A, *P* > 0.05). Fertile fish muscle exhibited a small, but statistically significant increase in the protein content compared to sterile fish; 20.6 ± 0.16% and 20.1 ± 0.16%, respectively (Fig. 1B, *P* < 0.05). In fish from the same study as in the current report, no differences in separable muscle based on eviscerated body weight were observed between fertile and sterile fish in December and January, but did decrease by 4% in 2N fish by March when most of the fish were at spawning (Aussanasuwannakul et al. 2011). In addition, there was no effect of age on muscle protein concentration, and only a transient effect on muscle fat, in fertile fish in this study when all time points were analyzed, including March samples, suggesting the high nutritional plane limited muscle protein degradation and fat mobilization in these fish even at spawning. Previous studies showed that female rainbow trout at full sexual maturation prior to spawning had a pronounced decrease (11%) in separable muscle and protein content (Salem et al. 2006b, 2008, 2010a). Nevertheless, the fish in the previous study only reached about 500 g at spawning (Salem et al. 2006a), whereas the fish in this study reached over 3000 g at spawning (Aussanasuwannakul et al. 2011) supporting differences in feeding between the studies.

Muscle crude fat content was lower for fertile fish compared to sterile fish; 7.8 ± 0.63% and 10.8 ± 0.61%; respectively (Fig. 1C, *P* < 0.01). In rainbow trout, gonadal development adversely affects energy homeostasis and thereby lowers muscle quality as a result of protein and lipid mobilization to support gonadogenesis (Salem et al. 2006a,b, 2010a). In this study, gravid fish muscle exhibited a slight increase in protein content compared to sterile fish when only December and January samples were analyzed; conversely, muscle fat content was greater in sterile fish compared to fertile fish. As just mentioned, we previously reported fat content was slightly altered in fertile fish over the course of gonad development (18% decrease between September and November) when fish are on a high plane of nutrition (Aussanasuwannakul et al. 2011). Nevertheless, the fat content of the sterile fish increased during maturation resulting in greater intramuscular fat in the sterile than the fertile fish at spawning, supporting lipid mobilization or utilization is increased in maturing fertile fish (Aussanasuwannakul et al. 2011). We previously reported that, at full sexual maturation, muscle protein content was negatively affected by sexual maturation and, there was no effect on fat content (Salem et al. 2006a). These results suggest that rainbow trout, at intermediate stages (2–3 months before spawning) of sexual maturation, likely mobilize intramuscular fat rather than protein to support gonadal maturation and changes in fat and protein are affected by nutritional plane. Previous studies showed that fish mobilize lipids from muscular tissues during sexual maturation although the majority derives from mobilization of visceral lipid stores (Nassour and Léger 1989; Aussanasuwannakul et al. 2011; Manor et al. 2012).

The decrease in muscle crude fat content of fertile fish was associated with a concomitant increase in the moisture content. The muscle of fertile fish contained 70.2 ± 0.53% moisture, compared to 67.7 ± 0.52% for sterile fish (Fig. 1D, *P* < 0.01). Muscle shear force, an indication of fillet firmness was higher (Fig. 1E, *P* < 0.01) in fertile than in sterile fish; 296.8 ± 11 g/g (BW) in fertile fish and 231.3 ± 11.2 g/g (BW) in sterile fish. The negative correlation between fat content and shear force may be explained by the fact that fat would provide lubrication during shearing and soften cooked fillets (Aussanasuwannakul 2011).

**Figure 2:**
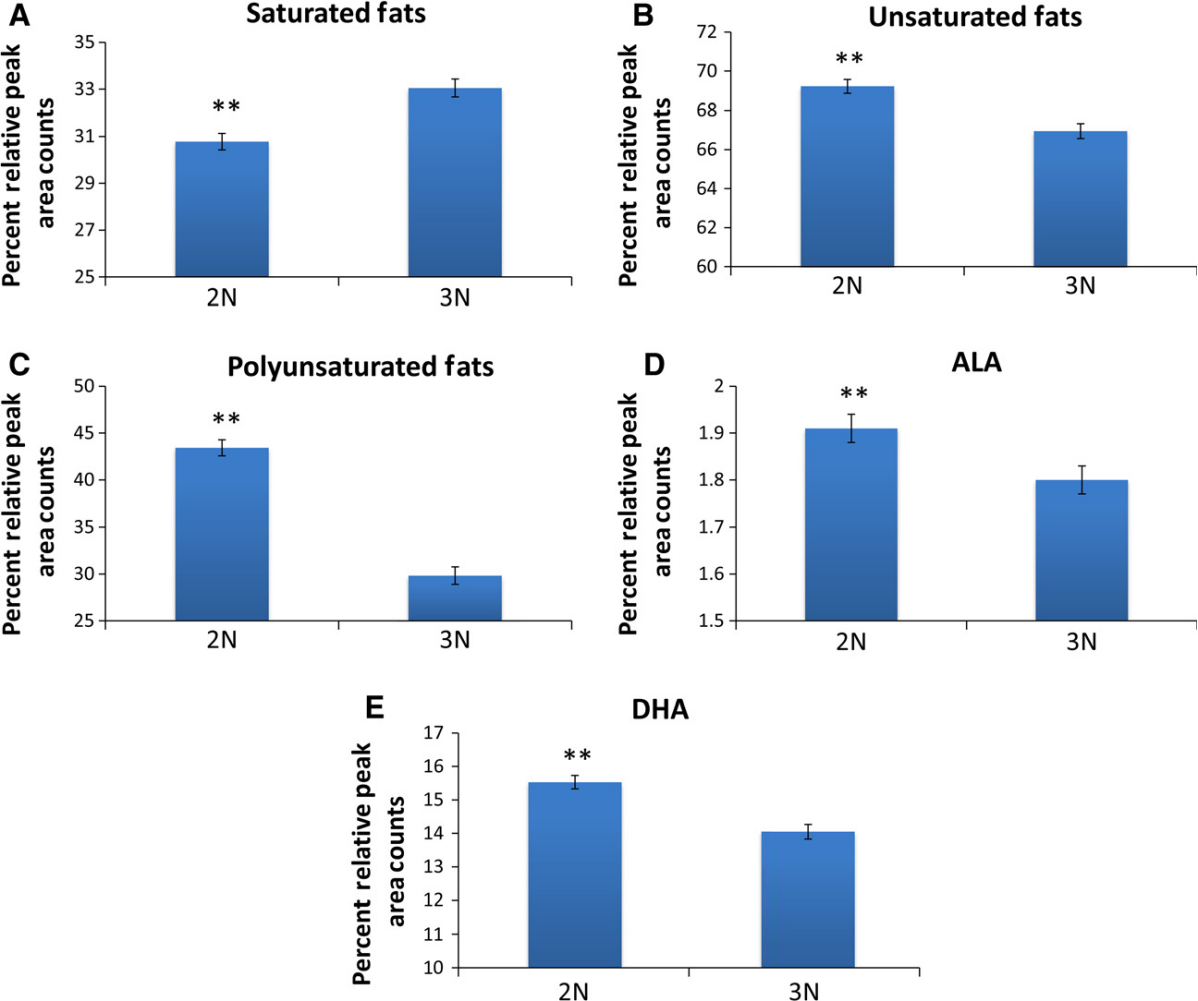
Effects of sexual maturation on rainbow trout relative amounts of the total saturated fatty acids (A), total unsaturated fatty acids (B), polyunsaturated fatty acids (C), alpha-linolenic acid [ALA] (D) and docosahexaenoic acid [DHA] (E). * and ** indicate the *P* values <0.05 and <0.01, respectively (LSMeans + SEM, *N* = 10). 2N and 3N indicate diploid and triploid fish, respectively.

Fillets from gravid fish contained smaller relative amounts of total saturated fatty acids compared to sterile fish; 30.78 ± 0.35 and 33.05 ± 0.38, respectively (Fig. 2A, *P* < 0.01). Conversely, muscle of gravid fish contained greater relative amounts of total unsaturated fatty acids compared to sterile fish; 69.22 ± 0.35 and 66.95 ± 0.38, respectively (Fig. 2B, *P* < 0.01). There was no difference in the monounsaturated fatty acids (data reported in Manor et al. 2012); however, gravid fish contained greater relative amounts of polyunsaturated fatty acids (43.43 ±0.86%) compared to sterile fish (29.83 ± 0.93%) (Fig. 2C, *P* < 0.01). In addition, gravid fish muscle contained greater amounts of two omega-3 fatty acids compared to sterile fish: (1) Alpha-linolenic acid (ALA); 1.91 ± 0.03% and 1.8 ± 0.03%, respectively (Fig. 2D, *P* < 0.01), and (2) docosahexaenoic acid (DHA); 12.63 ± 0.49% and 10.18 ± 0.53%, respectively (Fig. 2E, *P* < 0.01). The omega-3 fatty acid, eicosapentaenoic (EPA), was not affected (*P* > 0.05) by ploidy. These results support findings from a more comprehensive study in our laboratory which demonstrated that maturation affects fillet quality, especially muscle composition and texture. Changes in these quality attributes are caused by changes in total fat and fatty acid content (Aussanasuwannakul et al. 2011; Manor et al. 2012). The same study indicated that muscle of fertile fish accumulates fat up to 18 months; after this time, dietary energy is shifted from intramuscular fat deposition to ovarian growth (Manor et al. 2009; Aussanasuwannakul et al. 2011). Compared to unsaturated fats, saturated fats yield more energy; hence, saturated fats may be preferably catabolized over unsaturated fats (Manor et al. 2012). Changes in muscle fat content are also in agreement with a similar study by Nassour and Léger (1989) who showed that muscle fat is mobilized during latter stages of egg growth and continues after ovulation.

### Gene expression response to sexual maturation

**Figure 3:**
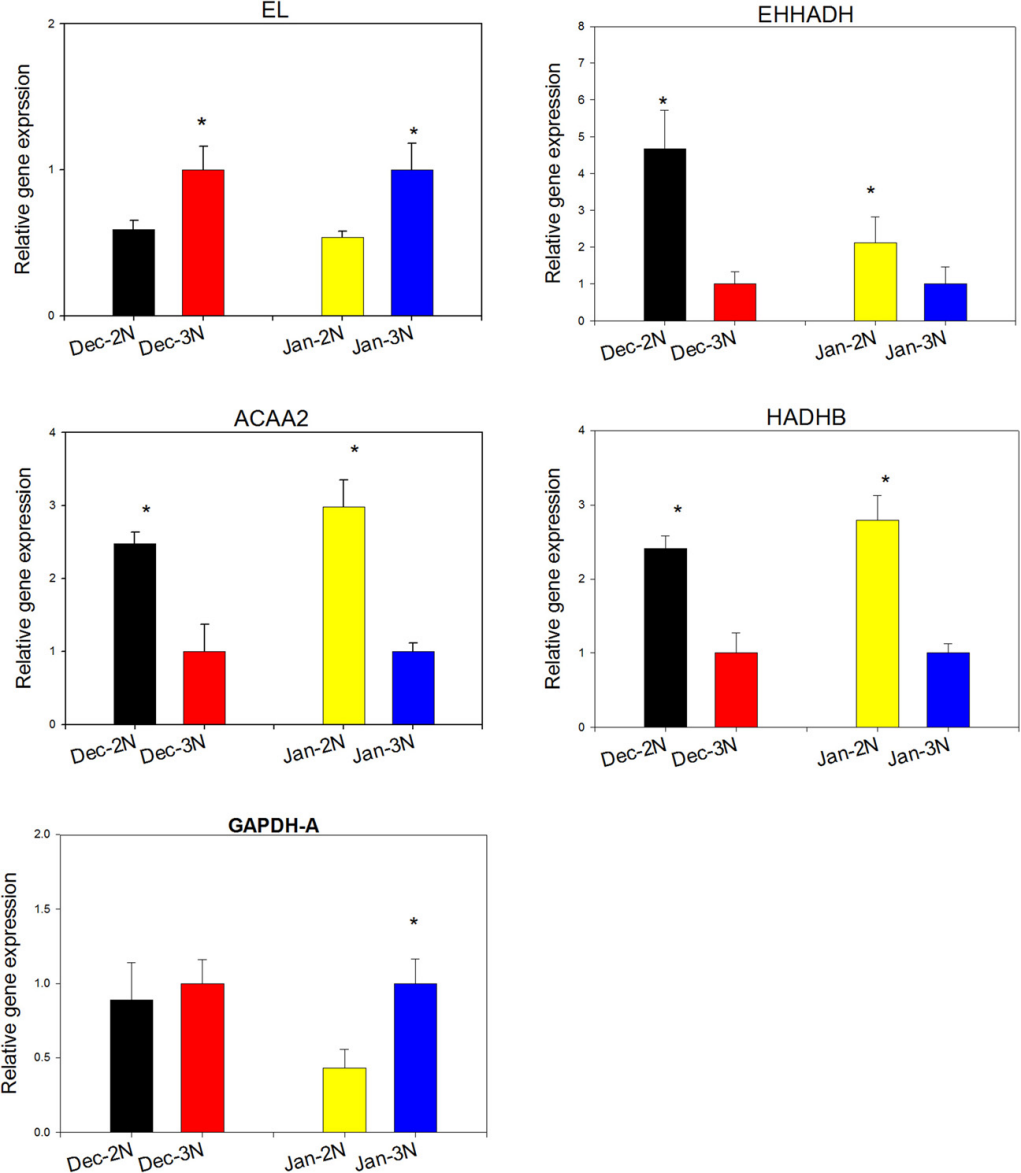
Quantitative real-time PCR confirmation of five genes identified by RNA-Seq as differentially expressed in muscle of gravid fish versus sterile fish. Differential expressions of four genes related to lipid metabolism (*el*, *ehhadh*, *acca2*, and *hadhb*) and an isoform of the gapdh gene were confirmed by qPCR. All genes showed statistically significant changes that are consistent with the RNA-Seq results (*P* < 0.05). * and ** indicate the *P* values <0.05 and <0.01, respectively, (LSMeans + SEM, *N* = 10). 2N and 3N indicate diploid and triploid fish, respectively.

Our recent studies showed that most of the effects of the sexual maturation on female rainbow trout growth rate, muscle growth, and quality starts to take place 2–3 months before spawning (Aussanasuwannakul et al. 2011; Manor et al. 2012). The gene expression signature of white (fast-twitch) muscle was profiled for gravid rainbow trout approaching spawning (2–3 months before spawning) and compared to the sterile (3N) trout profile. The RNA-Seq approach was used to help understand the mechanisms behind changes in muscle growth and quality at stages close to spawning. RNA-Seq identified 286 up-regulated and 358 down-regulated transcripts in gravid fish relative to sterile fish (FDR <0.01, ±twofold change) (Complete data set is available upon request from the corresponding author). KEGG metabolic pathway analysis of the differentially expressed genes revealed functional gene suites that suggest particular patterns of metabolic changes. Quantitative real-time PCR was used to validate expression of five genes identified by RNA-Seq as differentially expressed in gravid fish. Four of these genes (*el*, *ehhadh*, *acca2*, and *hadhb*, discussed below) were chosen according to their biological functions to confirm variation in lipid metabolism. All five genes showed statistically significant changes that are consistent with the RNA-Seq results (*P* < 0.05; Fig. 3).

#### Genes involved in lipid metabolism

**Figure 4:**
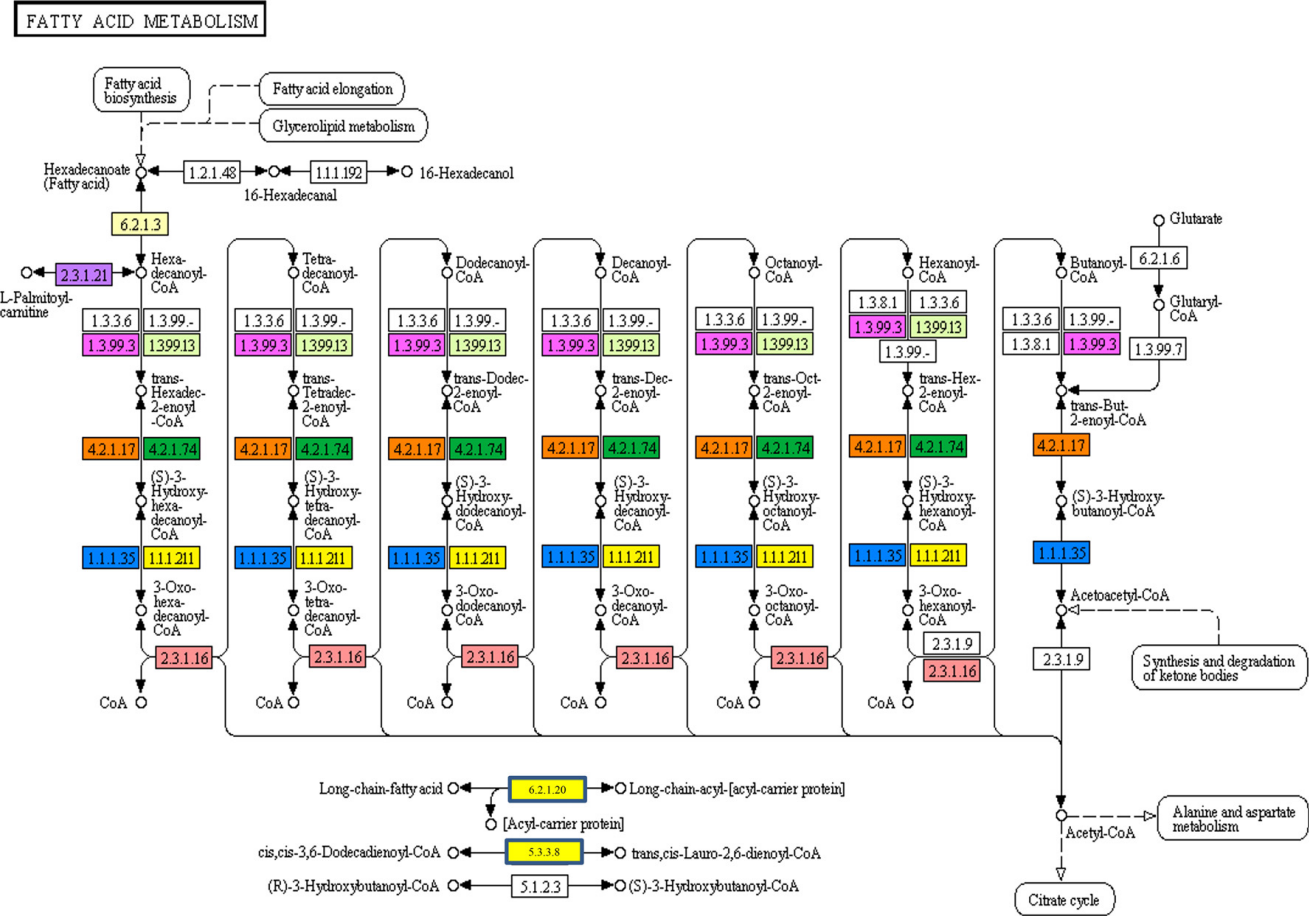
KEGG Reference pathway: colored boxes correspond to differentially expressed genes in muscle of gravid rainbow trout fish versus sterile fish that are assigned to the fatty acid metabolic pathway. Gene annotations and expression values are provided in Table 2.

RNA-Seq of gravid fish muscle showed 17 differentially expressed (16 up-regulated) transcripts involved in fatty acid metabolism. The list (Table 2 and Fig. 4) includes two transcripts encoding the mitochondrial carnitine palmitoyltransferase I alpha1a (*cpt1a*) gene that is located in the mitochondrial outer membrane and mediates the transport of long-chain fatty acids, thus initiating oxidation of these fatty acids (Lemas et al. 2012). Prolonged inhibition of *cpt1a* was reported to promote accumulation of muscle intramyocellular lipid in rats (Dobbins et al. 2001). A rainbow trout line selected for lean muscle exhibited a significantly higher level of expression of hepatic *cpt1a* than a fat selected line (Kamalam et al. 2012). In addition, the list includes four up-regulated transcripts that encode (1) Acyl-CoA dehydrogenase (*acdh-11*); (2) two transcripts similar to the Very Long-Chain Acyl-CoA Dehydrogenase (*vlcad* and *acadvl*) that act on 12–18 C fatty acids; and (3) one transcript similar to Medium-chain specific acyl-CoA dehydrogenase (*acadm*). Acyl-CoA dehydrogenases catalyze the first step of fatty acid β-oxidation in the mitochondria (Thorpe and Kim 1995). In cattle, *vlcad* genes were negatively correlated with intramuscular fat content (Jeong et al. 2012).

**Table 2:** Differentially expressed genes involved in fatty acid metabolism.

GenBank acc#	Enzyme	Gene symbol	Fold change 2N/3N	FDR *P* value	Enzyme Id
EZ812849	Mitochondrial carnitine palmitoyltransferase I alpha1a	CPT1A	3.7	1.8E-04	EC:2.3.1.21
EZ905317	Mitochondrial carnitine palmitoyltransferase I alpha1a	CPT1A	4.4	1.0E-07	EC:2.3.1.21
EZ896350	Acyl-CoA Dehydrogenase	ACDH-11	5.3	1.1E-03	EC:1.3.99.3
EZ905465	Very long-chain acyl-CoA synthetase (s27a2)	VLCAD	7.2	1.2E-03	EC:6.2.1.–6.2.1.3
EZ911051	Acyl-CoA dehydrogenase, very long chain	ACADVL	2.9	9.7E-08	EC:1.3.99
EZ763374	Medium-chain specific acyl-CoA dehydrogenase	ACADM	2.9	1.5E-05	EC:1.3.99.3
EZ906059	HADHB trifunctional protein, beta subunit	HADHB	2.5	1.8E-04	EC:2.3.1.16
EZ764956	Acetyl-CoA acyltransferase 2	ACAA2	2.8	1.9E-04	EC:2.3.1.16
EZ791335	Acetyl-CoA acyltransferase 2	ACAA2	3.7	8.1E-34	EC:2.3.1.16
BX305333	Fatty acid-binding protein 3, muscle and heart	FABP3	3.4	1.8E-23	
BX913099	Choline-phosphate cytidylyltransferase A	PCYT1A	2.0	2.9E-03	EC:2.7.7.15
EZ838632	EHHADH enoyl-CoA, hydratase/3-hydroxyacyl-CoA dehydrogenase/Peroxisomal bifunctional enzyme (echp)	EHHADH	5.8	1.7E-20	EC:4.2.1.171.1.1.355.3.3.8
FP319860	Peroxisomal trans-2-enoyl-CoA reductase	PECR	3.6	2.3E-05	EC: 1.3.1.38
EZ811897	1-acylglycerol-3-phosphate O-acyltransferase	ABHD5	13.3	4.0E-03	EC:2.3.1.51
EZ905495	Acyl-CoA dehydrogenase family, member 9	ACAD9	2.3	2.5E-11	EC:1.3.99
EZ770803	Monoacylglycerol lipase ABHD12	ABHD12	5.5	1.5E-03	EC:3.1.1.2
CU063634	Endothelial lipase	EL	−4.3	0.0E+00	EC:3.1.1.3
CU072692	Acetyl-CoA carboxylase/biotin carboxylase	ACACB	2.5	2.4E-09	EC:6.4.1.2–6.3.4.14

In gravid trout muscle, there were three additional up-regulated transcripts encoding enzymes that catalyze late steps in mitochondrial fatty acid oxidation; (1) beta subunit of the hydroxyacyl-CoA dehydrogenase/3-ketoacyl-CoA thiolase/enoyl-CoA hydratase (Hadhb) of the mitochondrial trifunctional protein multienzyme complex; and (2) Two different transcripts similar to acetyl-CoA acyltransferase 2 (*acaa2*). Activity of the Had enzyme was up-regulated in liver, but not in muscle, of fish fed a high-energy diet (Kolditz et al. 2008a). Moreover, gravid fish exhibited increased expression of a transcript related to fat metabolism; Choline-phosphate cytidylyltransferase A (Pcyt1a) that catalyzes a rate-controlling step in the biosynthesis of phosphatidylcholine (Wang et al. 2005) and fatty acid-binding protein 3 (Fabp3) that is thought to participate in the uptake, intracellular metabolism and/or transport of long-chain fatty acids (FABP3 Gene 2013).

In addition to mitochondrial fatty acid metabolic genes, gravid fish exhibited up-regulated expression of two transcripts encoding enzymes belonging to the peroxisomal fatty acid metabolic pathways; (1) peroxisomal bifunctional enzyme enoyl-CoA, hydratase/3-hydroxyacyl-CoA dehydrogenase (Ehhadh), one of four enzymes in the peroxisomal fatty acids β-oxidation pathway (Gene [Internet] 2004a), and (2) NADPH-specific peroxisomal trans-2-enoyl-CoA reductase (Pecr), the key enzyme for peroxisomal chain elongation pathway of fatty acids (Das et al. 2000). According to KEGG database, Pecr belongs to the biosynthesis of unsaturated fatty acids pathways (KEGG 2012). Up-regulation of the *pecr* gene may explain the increased unsaturated fatty acid content in gravid fish muscle compared to sterile fish (Fig. 2).

Regarding regulation of fatty acid metabolism, gravid fish muscle exhibited three up-regulated genes associated with increased fatty acid degradation: One gene is the 1-acylglycerol-3-phosphate O-acyltransferase gene (*abhd5*) that activates adipose triglyceride lipase (Atgl); Atgl is the rate-limiting enzyme in lipolysis. Mutations in this gene have been associated with a triglyceride storage disease (Chanarin-Dorfman syndrome) and impaired long-chain fatty acid oxidation (Lass et al. 2006). A second up-regulated gene is a transcript similar to acyl-CoA dehydrogenase (*acad9*) which is localized in the mitochondria and catalyzes a rate-limiting step in fatty acids β-oxidation. *Acad9* encodes for an enzyme that is especially active toward palmitoyl-CoA and long-chain, unsaturated substrates (Zhang et al. 2002; Gene [Internet] 2004b). The third up-regulated gene is monoacylglycerol lipase (Abhd12) that hydrolyzes intracellular triglyceride stores to fatty acids and glycerol in adipocytes (Gene [Internet] 2004c). In addition, gravid fish possessed a single gene with down-regulated expression; Endothelial lipase (El). El is a phospholipase that was suggested to represent an alternative mechanism to lipoprotein lipase, believed to be the only enzyme accountable for the catabolism of triglycerides (Kratky et al. 2005). Adipocytes that lacked lipoprotein lipase showed up-regulated expression of El gene in mice (Kratky et al. 2005). However, gravid fish exhibited a single gene with marginal (2.5-fold) up-regulated expression, suggesting limited fatty acid degradation; this gene encodes a subunit of acetyl-CoA carboxylase (Acacb) multifunctional enzyme complex. Acac catalyzes carboxylation of acetyl-CoA to malonyl-CoA, thus controlling fatty acid oxidation in muscle. In lipogenic tissues such as liver, Acac controls the rate-limiting step of long chain fatty acid biosynthesis. This enzyme activity can be controlled at the transcriptional level (Tong and Acetyl-coenzyme 2005). Changes in *acacb* expression may represent a temporary effect rather than a long-term change.

Collectively, differential expression of the aforementioned genes suggests enhanced fatty acid breakdown, via β-oxidation, in the mitochondria and peroxisome. Up-regulation of the β-oxidation pathways may explain the decreased fat content of gravid fish muscle compared to sterile fish (Fig. 1). To meet the increasing energetic demand of ovarian growth, up-regulated, β-oxidation mechanisms are coordinated in muscle, a significant tissue in fish for overall fatty acid catabolism (Nanton et al. 2003). This assertion is partially supported by reports showing that rainbow trout selected for fat muscle had reduced fatty acid oxidation in the liver, but not in muscle, compared to a lean selected line (Kolditz et al. 2008a,b).

#### Genes involved in glucose metabolism

Muscle of gravid rainbow trout had six differentially expressed transcripts belonging to the glycolysis metabolic pathway (Table 3). Three transcripts exhibiting up-regulated expression were: (1) Phosphoglucomutase (Pgm) that catalyzes the interconversion of glucose 1-phosphate and glucose 6-phosphate and thus plays an important role in glycogenolysis/glycogenesis; (2) 6-phosphofructokinase (Pfk) that catalyzes the first irreversible, and thus the most important regulatory step in glycolysis; and (3) Pyruvate kinase (Pk) that potentiates the last and one of the most important regulatory steps of the glycolytic pathway. In contrast, a transcript similar to enolase (Eno), which catalyzes conversion of 2-phosphoglycerate to phosphoenolpyruvate (a later step in glycolysis), exhibited down-regulated expression. In addition, two transcripts encoding glyceraldehyde 3-phosphate dehydrogenase (Gapdh) showed conflicting expression (one up- and one down-regulated, accession no. EZ796055 and EZ905446 respectively.

**Table 3:** Differentially expressed genes involved in glucose metabolism citric acid cycle, oxidative phosphorylation and pentose phosphate pathway.

GenBank acc#	Enzyme	Gene symbol	Fold change 2N/3N	FDR *P* value	Enzyme Id
Glucose metabolism					
CA354174	Phosphoglucomutase	PGM	16.7	2.5E-04	EC:5.4.2.2
EZ767419	6-phosphofructokinase	PFK	5.0	1.1E-14	EC:2.7.1.11
EZ899830	Pyruvate kinase	PK	3.1	5.1E-09	EC:2.7.1.40
EZ796055	Glyceraldehyde-3-phosphate dehydrogenase	GAPDH	4.6	1.1E-03	EC:1.2.1.12
EZ905446	Glyceraldehyde-3-phosphate dehydrogenase	GAPDH	−2.7	9.2E-04	EC:1.2.1.12
EZ906644	Phosphopyruvate hydratase (enolase)	ENO	−2.4	8.4E-03	EC:4.2.1.11
EZ778529	Pyruvate dehydrogenase (acetyl-transferring)	PDH	9.8	1.2E-08	EC:1.2.4.1
EZ905434	Aldehyde dehydrogenase [NAD(P)+]	ALDH	−2.1	3.1E-06	EC:1.2.1.5
CU063745	Alcohol dehydrogenase	ADH	−2.1	0.0E+00	EC:l.l.l.l
Citrate cycle (TCA cycle)					
EZ770794	Citrate (Si)-synthase	CS	2.1	3.5E-03	EC:2.3.3.1
EZ786023	ATP citrate synthase	ACLY	2.5	9.5E-03	EC:2.3.3.8
FP320359	Citrate (pro-3S)-lyase	citF	2.6	7.3E-04	EC:4.1.3.6
FP322783	Succinate dehydrogenase (ubiquinone)	SDH	2.2	3.0E-20	EC:1.3.5.1
EZ786023	Succinate-CoA ligase (GDP-forming)	LSC	2.5	9.5E-03	EC:6.2.1.4
EZ786023	Succinate-CoA ligase (ADP-forming)	LSC	2.5	9.5E-03	EC:6.2.1.5
EZ783355	Aconitate hydratase	ACO	2.4	1.0E-09	EC:4.2.1.3
Oxidative phosphorylation					
EZ763519	NADH:ubiquinone reductase (H+-translocating)	NDH	2.3	8.1E-03	EC:1.6.5.3
AF465782	Ubiquinol-cytochrome-c reductase	UCRI	3.0	1.1E-06	EC:1.10.2.2
GE830294, BX870902	Cytochrome-c oxidase	COX	2.7	2.9E-03	EC:1.9.3.1
BX318194	H+-exporting ATPase	ATPase	2.1	7.5E-03	EC:3.6.3.6
Pentose phosphate pathway					
EZ779245	Phosphogluconate dehydrogenase (decarboxylating)	GND	−3.3	3.3E-03	EC:1.1.1.44
EZ779245	Phosphogluconate 2-dehydrogenase	PGDH	−3.3	3.3E-03	EC:1.1.1.43
EZ795956	Ribose-phosphate diphosphokinase	PRPS	−2.2	3.4E-04	EC:2.7.6.1

Moreover, gravid fish had three differentially expressed transcripts belonging to the pyruvate metabolic pathway (Table 3). A transcript similar to pyruvate dehydrogenase (*pdh*) was up-regulated. This enzyme connects the glycolysis pathway to the citric acid cycle by converting pyruvate into acetyl-CoA. In contrast, two transcripts, aldehyde dehydrogenase (*aldh*) and alcohol dehydrogenase (*adh*), were down-regulated in gravid fish muscle. Adh enzymes metabolize a wide variety of substrates, including alcohols and lipid peroxidation products. Functional Adh enzymes are expressed in zebra fish (Reimers et al. 2004); however, studies concerning functions of Adh and Aldh have been limited in fish (Lassen et al. 2005).

Overall, gravid fish, 2–3 months before spawning, exhibited five up- and four down-regulated transcripts involved in glucose metabolism. In contrast, fish at full maturity have shown consistent decreased expression (measured at transcriptional and proteomic levels) of genes encoding enzymes of the glycolysis pathway, indicating a reduction in glucose utilization for atrophying trout muscle during sexual maturation (Salem et al. 2006b, 2008, 2010a). The reduced transcription of genes involved in glucose metabolism was not observed at intermediate stages of sexual maturity; perhaps due to the elevated nutritional plane used throughout this study. Reduction in glucose metabolism is a common characteristic of muscle atrophy associated with many experimental and systemic diseases in mammals and fish (Lecker et al. 2004; Raffaello et al. 2006; Salem et al. 2006b). The contradicting expressions of two *gapdh* transcripts may represent differential gene expressions of two different isoforms. Further studies are needed to distinguish potential *gapdh* isoform-specific differential gene expression.

#### Genes involved in the citric acid cycle, oxidative phosphorylation and pentose phosphate pathway

Gravid rainbow trout muscle possessed increased expression of seven transcripts from within the citric acid cycle (TCA). These transcripts were Citrate (Si)-synthase (*cs*), ATP citrate synthase (*acly*), Citrate (pro-3S)-lyase (*citF*), Succinate dehydrogenase (*sdh*), two transcripts similar to succinate-CoA ligase (*lsc*), and Aconitate hydratase (*aco*) (Table 3). In addition, four genes involved in oxidative phosphorylation were up-regulated in the gravid fish muscle. These genes were: (1) NADH: ubiquinone reductase (H+-translocating) (*ndh*); (2) Ubiquinol-cytochrome-c reductase (*ucri*); (3) two transcripts similar to cytochrome-c oxidase (*cox*); and (4) H+-exporting ATPase. Furthermore, gravid fish muscle exhibited decreased accumulation of three transcripts belonging to the Pentose phosphate pathway: (1) Phosphogluconate dehydrogenase (*gnd*); (2) Phosphogluconate 2-dehydrogenase (*pgdh*); and (3) ribose-phosphate diphosphokinase (*prps*) (Table 3). The increased expressions of TCA and oxidative phosphorylation genes is explained by increased fatty acid oxidation that generates acetyl-CoA for entry into the TCA cycle and leads to increased ATP production through oxidative phosphorylation. The enhanced expression of TCA and oxidative phosphorylation genes is consistent with our previous microarray studies that showed enhanced capacity of gravid fish, compared to sterile fish, for aerobic respiration (Salem et al. 2006b, 2008). Similar to the gravid females of this study, salmon, during spawning, switched from anaerobic glycolysis to oxidative phosphorylation (Miller et al. 2009).

#### Genes involved in amino acid metabolism

Muscle of gravid rainbow trout had seven differentially expressed transcripts belonging to amino acid metabolic pathways (Table 4). KEGG pathway mapping analysis clustered these seven transcripts into three specific pathways.

- The glycine, serine, and threonine metabolic pathway had four up-regulated transcripts encoding; (1) phosphoglycerate dehydrogenase (*phgdh*); this is the first and rate-limiting enzyme that controls flux from glycolysis into the serine biosynthesis (Mullarky et al. 2011), (2) cystathionine beta-synthase (*cbs*); this enzyme converts homocysteine and serine to a cystathionine, which is convertible to the amino acid cysteine (Genetics Home Reference 2013), (3) L-threonine 3-dehydrogenase (*tdh*; this enzyme acts in the degradation of threonine to glycine (KEGG 2012) and (4) choline dehydrogenase (gbsB); this enzyme is involved in the glycine betaine biosynthetic process from choline (UniProt Consortium 2013).
- The arginine and proline metabolic pathway had two up-regulated and one down-regulated transcripts. (1) argininosuccinate lyase (*asl*) was up-regulated, it catalyzes the conversion of argininosuccinate to arginine that is later metabolized to urea and ornithine (Genetics Home Reference 2013). Although the urea cycle occurs mainly in liver cells, the presence of all urea cycle enzymes in muscle was previously reported in alkaline-adapted tilapia (Anderson and Wright 2001). (2) A transcript encoding a bifunctional enzyme that catalyzes proline biosynthesis and consists of two domains, an N-terminal glutamate 5-kinase domain (proB) and a C-terminal glutamate-5-semialdehyde dehydrogenase (*pro2*) domain (InterPro 2013), was up-regulated. (3) A transcript encoding an aminoacylase (*acy*) that is involved in hydrolysis of N-acylated or N-acetylated amino acids to amino acids and an acyl group was down-regulated (Genetics Home Reference 2013).
- The alanine, aspartate and, glutamate metabolism pathway had two differentially expressed transcripts. These transcripts were: (1) a down-regulated transcript encoding carbamoyl-phosphate synthase (*cad*) that catalyzes the production of carbamoyl phosphate from glutamine (Thoden et al. 2002). (2) An up-regulated transcript similar to the aforementioned *asl*.

**Table 4:** Differentially expressed genes involved in amino acid metabolism.

GenBank acc#	Enzyme	Gene symbol	Fold change 2N/3N	FDR *P* value	Enzyme Id
Glycine, serine and threonine metabolism					
EZ779245	Phosphoglycerate dehydrogenase	PHGDH	3.3	3.27E-03	EC:1.1.1.95
EU315112	Cystathionine beta-synthase	CBS	2.4	4.65E-03	EC:4.2.1.22
EZ788023	L-threonine 3-dehydrogenase	TDH	2.3	1.82E-03	EC:1.1.1.103
CU063745	Choline dehydrogenase	gbsB	2.1	0.00E+00	EC:l.l.l.l
Arginine and proline metabolism					
EZ797984	Argininosuccinate lyase	ASL	2.5	9.05E-03	EC:4.3.2.1
EZ875727	Glutamate 5-kinase	proB	4.9	9.38E-04	EC:2.7.2.11
EZ875727	Glutamate-5-semialdehyde dehydrogenase	PR02	4.9	9.38E-04	EC:1.2.1.41
EZ767068	Aminoacylase	ACY	−2.9	2.22E-11	EC:3.5.1.14
Alanine, aspartate and glutamate metabolism					
EZ782724	Carbamoyl-phosphate synthase	CAD	−2.7	1.33E-03	EC:2.1.3.2
EZ797984	Argininosuccinate lyase	ASL	2.5	9.05E-03	EC:4.3.2.1

Muscle protein content was minimally increased in gravid fish compared to sterile fish; 20.6 ± 0.16% and 20.1 ± 0.16%, respectively. It is also possible that the greater intramuscular lipid in sterile fish diluted proteins. Atrophying muscle of fertile fish at full maturity, which had 11% less protein content compared with nonatrophying muscle of sterile fish (Salem et al. 2006b), exhibited down-regulation of myofibrillar protein genes and up-regulation of catheptic proteolysis (Salem et al. 2006a,b). In this study, the differential expression of genes associated with amino acid metabolism is consistent with our observation that protein content was minimally affected by ploidy during mid-late vitellogenesis. Some of the differentially expressed genes involved in amino acid metabolism, perhaps, represent intermediary metabolic changes.

## Conclusion

Female rainbow trout, 2–3 months prior to spawning, mobilized intramuscular fat rather than proteins to fuel gonadal maturation; whereas, protein, which is mobilized at terminal stages of sexual maturation when dietary nutrients are limiting and body stores of fat are low (Salem et al. 2006a,b), was minimally changed. RNA-Seq identified a gene expression signature that is consistent with biochemical changes in gravid fish and sterile fish. More transcripts associated with metabolic pathways of fatty acid degradation and biosynthesis of the polyunsaturated fatty acids were present in gravid females. Polyunsaturated fatty acids are important for fish egg quality (Yanes-roca et al. 2009) and we observed changes in fatty acid profiles during maturation in these fish (Manor et al. 2012). This transcriptomic signature is different from the signature observed for fish at terminal stages of sexual maturation when nutrient and energy reserves are limited. The later stages included decreased expressions of genes involved in cell growth, anaerobic respiration, and protein biosynthesis and increased expression of proteases (Salem et al. 2006a,b, 2010a).
